# Neonates’ responses to repeated exposure to a still face

**DOI:** 10.1371/journal.pone.0181688

**Published:** 2017-08-03

**Authors:** Emese Nagy, Karen Pilling, Rachel Watt, Attila Pal, Hajnalka Orvos

**Affiliations:** 1 Psychology, University of Dundee, Park Place, Dundee, DD14HN, United Kingdom; 2 Department of Obstetrics and Gynaecology, University of Szeged, Szeged, Hungary; Universite de Bretagne Occidentale, FRANCE

## Abstract

**Aim:**

The main aims of the study were to examine whether human neonates’ responses to communication disturbance modelled by the still-face paradigm were stable and whether their responses were affected by their previous experience with the still-face paradigm.

**Methods:**

The still face procedure, as a laboratory model of interpersonal stress, was administered repeatedly, twice, to 84 neonates (0 to 4 day olds), with a delay of an average of 1.25 day.

**Results:**

Frame-by-frame analysis of the frequency and duration of gaze, distressed face, crying, sleeping and sucking behaviours showed that the procedure was stressful to them both times, that is, the still face effect was stable after repeated administration and newborns consistently responded to such nonverbal violation of communication. They averted their gaze, showed distress and cried more during the still-face phase in both the first and the second administration. They also showed a carry-over effect in that they continued to avert their gaze and displayed increased distress and crying in the first reunion period, but their gaze behaviour changed with experience, in the second administration. While in the first administration the babies continued averting their gaze even after the stressful still-face phase was over, this carry-over effect disappeared in the second administration, and the babies significantly increased their gaze following the still-face phase.

**Conclusion:**

After excluding explanations of fatigue, habituation and random effects, a self-other regulatory model is discussed as a possible explanation for this pattern.

## Introduction

The still-face paradigm (SFP) [1] is a widely used laboratory method to assess young infants’ behavioural responses to a simulated, major violation of interpersonal communication. The procedure involves an experimenter or the mother first engaging a young infant and then unexpectedly becoming unresponsive, as if freezing in the middle of a conversation, posing motionless with a still face. The procedure is robust, and the main still-face effect is characterised by striking behavioural changes. Infants withdraw, avert their gaze, display negative affect, become increasingly distressed, start crying, and smile less during the still-face manipulation compared with the baseline engagement period [1–3]. The second characteristic of the response is a spill-over or carry-over [1, 4] or reunion effect [5], in which the behavioural responses to the still face continue beyond the manipulation phase. Even after the experimenter resumes communication with the infant, the infant continues averting its gaze from the experimenter, stays distressed and, generally, fails to re-engage with the experimenter on a pre-manipulation level [1, 3].

Whether responses to the SFP are stable or change with development have been debated [6]. Gusella, Muir and Tronick [7], Shapiro et al. [8] and Toda and Fogel [9] found no age-related changes in the response between three- and six-months of age. Similarly, Striano and Rochat [10] and Lamb et al. [11] reported a stability of the still-face response between one to seven and seven to ten months of age, respectively. Lamb et al. [11] and Mesman et al. [6] also suggested that the effect is considerably stable, although the intervals between the points of testing in the above studies are too large and the number of studies are too few for a definite conclusion.

Cosette et al. [12], however, found stability in positive emotional responsivity in their longitudinal study with infants between 2.5 and 5 months of age. Fine-grained analyses by Moore, Cohn and Campbell [13] found an increase in gaze aversion from two to six months of age. Melinder et al. [14] found more overall negative responses in younger infants at two and four months of age when compared with six and eight month olds.

In their review, Mesman, van Ijzendoorn and Bakermans-Kranenburg [6] concluded that the effect peaks around three months of age, but infants as young as six weeks [15] and two months [16, 17] decreased eye contact and showed increased negative affect in response to the still face.

Whether the still-face response appears around six weeks of age or is present from as early as the first day of life has been addressed in three experiments. Bertin and Striano [15] did not find the still-face effect to be present in newborns. Bigelow and Power [18] also did not find it in 28 mother–infant dyads with enhanced skin-to-skin contact intervention and 52 mother–infant dyads in the control group. They found the still-face response to appear at one month of age in the intervention group but not until three months of age in the control group. Nagy [3], however, reported that newborn infants between 3 and 96 hours after birth were able to adjust their behaviours according to the social responsiveness of their interaction partner during the still-face paradigm, as older infants do. Newborns in that study decreased eye contact, showed distressed faces and cried more during the procedure compared with the baseline and a control group. After the experimenter resumed responsiveness, the newborns displayed carry-over effects, continuing to avert their gaze and showing further increased distress and crying. Newborns in the control group who were engaged over a comparable length of time without still-face manipulation showed none of these behavioural changes.

Given newborn infants’ limited experience with social interaction and that the data from Nagy [3] is the only research confirming newborns’ responsivity to the SFP, the current research aimed to explore whether neonates’ responses to the procedure were stable. This aim was achieved by repeatedly exposing newborn infants to SFP at two different times.

Because newborns’ responses are analogous [3] to those of older infants [1], it has been assumed that the meaning of the still-face situation is the same for newborns. However, given the contradictory findings in the literature, this is not necessarily the case, and it is possible that the reaction is not stable.

The present study first had to confirm whether the still-face effect exists in neonates. If neonates displayed the still-face effect, their avoidance would be measured by gaze aversion and their distress would be measured by the existence of a distressed face and crying. In addition, some of the effects would continue to carry over beyond the still-face phase into the reunion phase [3].

Second, the stability of the responses was tested through a subsequent administration of the SFP. It was hypothesized that if the disruption of communication in the SFP were experienced as a source of stress by the newborns, they would react with the same set of avoidance and stress responses each time the stress occurred. In this case, the responses to the SFP would be similar and similarly robust, as indicated by gaze aversion, increased facial distress and crying at both the first and the repeated administration.

Given that only one study found evidence for the still-face effect in neonatal infants [3] while two other studies found no evidence for it [15, 18], it was also possible that the still-face effect is situational in the neonate and likely to be unstable. In that case, there would be no consistent changes in their behaviour.

It was also possible that the meaning and significance of the social stress originating from the situation are dependent on social experience. In that case, newborns might react to the unexpected disruption in communication, but they would habituate when the procedure was administered a second time. If they habituated to the situation, the behavioural responses, gaze aversion, facial distress and crying might still increase during the first administration of the still-face phase, but they would significantly dampen in its second administration, and there would be a significantly less robust still-face effect when the behavioural responses to the still-face phase were cross-compared between the two administrations.

Alternatively, it was also possible that newborns would exhibit a stable stress response every time the SFP was administered but begin to show adaptive behavioural changes with the subsequent SFP. In this case, it would be expected that the newborns would continue to perceive the situation as stressful, and decreased eye contact and increased distressed face and crying would be comparable during the still-face situation in both the first and the second administration. The newborns’ responses during the reunion period, however, would show changes reflecting adaptive self-regulatory processes.

To address the above questions, the SFP was administered to neonatal infants twice in their first days of life, using the same design as in Nagy’s earlier experiment [3] and, for the purpose of comparability, using the same outcome variables. Infants’ gaze behaviour (i.e. looking at the experimenter) was coded to measure approach behaviours, and distress behaviours (i.e. distressed face and crying) and self-regulatory behaviours (i.e. sucking fingers, hands or mouth, as well as sleeping) were coded and analysed. First, the behavioural effects of the first administration were analysed to establish whether the newborns responded to the still-face effect manipulation. Then, the effect of the repetition was analysed, and finally, the effects of the two administrations were compared.

## Materials and methods

### Participants

The study took place over nine periods each lasting 7–10 days between 2006 and 2011, according to the availability of the researchers. Within these data collection periods, the researchers approached the mothers of healthy, singleton newborn infants to take part in the study. Mothers who had no obstetric complications and whose newborns were healthy and required no neonatal intensive care unit observation were invited to participate by the neonatologists on the neonatal ward. Of those, the newborns of mothers who signed an informed consent form were included in the study.

Eighty-four newborn infants (48 boys, 36 girls) were examined with the still face paradigm (SFP) twice. The average age of the babies was 1.33 days (SD = 1.54, range 0–4 days, the youngest 2 hours old) and they were born on average at 38.77 gestational weeks (SD = 1.19; 36–40 weeks) with an average weight of 3374 g (SD = 475g, 2040–4510 g). Thirty newborns were born by vaginal deliveries and 54 by caesarean section. All babies had 9 or above Apgar scores 10 minutes after birth. The study has been reviewed and approved by the Ethical Committees of the University of Dundee and the Albert Szent-Györgyi Medical University, Szeged. The data collection happened at the Neonatal Ward of the Obstetrics and Gynaecology Clinic at University of Szeged, while the coding and the analyses of the data at the University of Dundee, Scotland.

### Procedure

The examination was carried out in a separate room that was integral part of the Neonatal Ward. The room had constant illumination and a temperature of 28° C. Newborns were examined 30–90 minutes after feeding. Infants sat in a newborn seat placed on an examination table. The experimenter, who was the same throughout, stood in front of the infant, approximately 30 centimetres away. A Panasonic NVGS27B digital video-camera mounted on a tripod recorded the experiment, placed behind the experimenter. A mirror was placed behind the baby’s seat to ensure that the experimenter’s face was visible on the video-recordings when her face was not directly visible from the same camera angle as the newborn was recorded.

The study, similarly to the earlier study that established neonates’ responsivity to the SFP [3], and similarly to the original study reported by Tronick et al.’ [1] employed three 180-second length phases. The SFP started with a 3-minute interaction that included both verbal and non-verbal communication with the baby, (Phase 1: P1), followed by a 3-minute period when the experimenter became unresponsive and posed a neutral, still face, silently (Still Face: SF). Finally, the experimenter resumed the interaction with the baby (Phase 3: P3) for another 3 minutes. The procedure was repeated with the babies an average one day later (mean age at the second administration = 2.58 days SD = .1.33, 1 day later with 53 babies, 2–4 days later with 24 babies, and with 7 babies the delay was under 1 day, 2–5 hours). Statistical analyses showed that the length of the delay had no effect on any of the outcome variables therefore the sample was analyzed as one group.

#### Coding

Two independent coders who have not been involved in the design of the study, the data collection, and interpretation, coded the data. The three phases were coded frame-by-frame, using the Noldus Observer-Pro 5.0 system [19]. The behavioural variables coded in this study were identical to the variables coded in Nagy’s earlier experiment that found evidence for neonatal responsivity to the SFP [3]. We chose to code the same variables, thus the results of the two studies could be directly comparable. Infants’ gaze behaviour (looking at the experimenter) was coded as approach behaviour. Distress was coded using the codes of distressed face (distress on the face with or without vocalization and fussing but not crying), and crying (audible crying). Sucking the fingers, hands and mouth, and sleeping were coded as self-regulatory behaviours. Gaze and negative affective behaviours such as distress and crying are the most commonly coded variables in the SFP literature, therefore the results are also comparable to results with older infants [6]. While coding, the part of the screen showing the experimenter was covered for the coders, who were unaware of the experimenter’s behaviour. All behaviours were coded and analysed for both their frequencies and durations.

#### Reliability coding

Twenty-five percent of the data were reliability coded. Cohen’s kappas for inter-rater reliabilities of the frequency data in the P1 period ranged from .65-.94 with an average of .80, in the SF .65-.90 with an average of .75, in P3 .66-.95 with an average of .82.

#### Statistical analysis

Frequencies of the behaviours (rate/minute) calculated by the Observer XT-9.0 system [20] were used for statistical analysis. Repeated Analysis of Variances (ANOVAs) were conducted using SPSS 22.0 for Windows statistical software (SPSS, Inc., Chicago, IL), and a p < .05 was accepted as significant throughout. When Mauchley’s tests indicated a violation of the assumption of sphericity, degrees of freedom were corrected using Greenhouse-Geisser sphericity estimates. Post-hoc comparisons have been carried out using Bonferroni correction.

## Results

### 1. Do neonates respond to the SFP? Validation of the still face effect in the first administration of the SFP

Repeated Analysis of Variances (ANOVAs) were conducted to investigate the effect of the experimental phases (P1, SF, P3) on the change of the frequencies of the behaviours.

#### Gaze behaviour: Frequency and duration

There was a significant main effect of the experimental manipulation on the frequency of gaze (F_2,166_ = 16.07, p<0.001, ηp^2^ = .16). Pairwise comparisons showed that babies significantly decreased the frequency of the eye contact from P1 to SF, and eye contact remained significantly lower in P3 compared to P1 (See Table 1).

**Table 1:** Changes of the frequencies and durations of gaze, distressed face, crying and sleeping over P1, SF and P3 in the first administration, and results of the post-hoc analyses. Mean for Frequency: Rate/minute, for Duration: Seconds.

Behaviour	Measure	Phases (Mean/SE)			Post-hoc comparison		
		P1	SF	P3	P1-SF	SF-P3	P1-P3
**Gaze**	Frequency	6.70 (0.32)	5.38 (0.38)	4.89 (0.37)	< .001	.43	< .001
	Duration	16.88 (1.10)	12.68 (1.16)	10.72 (0.96)	.002	.16	< .001
**Distressed face**	Frequency	0.31 (0.05)	0.56 (0.09)	0.77 (0.12)	.006	.11	.001
	Duration	3.70 (1.10)	4.98 (1.10)	6.75 (1.25)	.99	.43	.036
**Crying**	Frequency	0.05 (0.03)	0.23 (0.06)	0.51 (0.12)	.003	< .001	.008
	Duration	0.39 (0.23)	6.74 (1.94)	8.04 (2.04)	.003	1.00	.001
**Sleeping**	Frequency	0.09 (0.03)	0.15 (0.05)	0.26 (0.07)	.70	.24	.078
	Duration	1.20 (0.56)	2.56 (0.91)	3.97 (1.06)	.55	.34	.064
**Sucking**	Frequency	1.66 (0.14)	1.79 (0.18)	1.72 (0.17)	1.00	1.00	1.00
	Duration	14.00 (1.52)	16.06 (2.09)	14.03 (1.43)	.84	.94	1.00

There was also significant main effect of the experimental manipulation on the duration of gaze (F_2,166_ = 15.48, p<0.001, ηp^2^ = .16). Pairwise comparisons showed that babies significantly decreased the duration of the eye contact from P1 to SF, had a tendency to further decrease the duration from SF to P3, and the eye contact remained significantly lower in P3 compared to P1 (See Table 1).

#### Distressed face: Frequency and duration

There was a significant main effect of the experimental manipulation on the frequency of the distressed face (F_1.46,135.56_ = 10.61, p<0.001, ηp^2^ = .11). Babies significantly increased the frequency of this behaviour from P1 to SF and then from SF to P3. The increase from P1 to P3 remained significant (See Table 1)

There was also a significant main effect of the experimental manipulation on the duration of the distressed face (F_2.166_ = 3.10, p = .048, ηp^2^ = .09). Babies significantly increased the duration of this behaviour from P1 to P3. The changes between P1 and SF and SF and P3 were not significant. (See Table 1)

#### Crying: Frequency and duration

There was a significant main effect on the frequency of crying (F_1.27,105.00_ = 13.27, p<0.001, ηp^2^ = .14) that increased from P1 to SF, and then from SF to P3. The frequency of crying was significantly higher in P3 compared to P1 (See Table 1).

There was a significant main effect on the duration of crying (F_2,166_ = 10.05, p<0.001, ηp^2^ = .11) that increased from P1 to SF, did not significantly change from SF to P3 and remained significantly higher in P3 compared to P1. (See Table 1).

#### Sleeping: Frequency and duration

There was a significant main effect on the frequency of sleeping (F_1.62,133.77_ = 3.84, p<0.05, ηp^2^ = .04). Babies significantly increased the frequency of this behaviour from P1 to P3 and had a tendency to increase it from the SF to P3. Sleeping in P1 and SF were comparable (See Table 1).

There was a significant main effect on the duration of sleeping (F_1.62,134.62_ = 3.75, p = 0.026, ηp^2^ = .043). Babies significantly increased the duration of this behaviour from P1 to P3. The change between P1 and SF and SF and P3 were not significant. (See Table 1).

#### Sucking: Frequency and duration

The frequency or the duration of the sucking (F_2,166_ = .26, n.s.; F_1.84,152.30_ = .43, n.s.) had not been affected by the experimental manipulation.

### 2. Do neonates respond to the second administration of the SFP?

Repeated Analysis of Variances (ANOVAs) were conducted to investigate the effect of the experimental phases (P1, SF, P3) on the change of the frequencies of the behaviours.

#### Gaze behaviour: Frequency and duration

There was a significant main effect of the experimental manipulation on the frequency of gaze (F_2,166_ = 8.11, p<0.001, ηp^2^ = .11). Pairwise comparisons showed that babies significantly decreased the frequency of the eye contact from P1 to SF, but significantly increased it from SF to P3. The frequencies in P1 and P3 were comparable. (See Table 2).

**Table 2:** Changes of the frequencies and durations of gaze, distressed face, crying and sleeping over P1, SF and P3 in the second administration with post-hoc analyses. Mean for Frequency: Rate/minute, for Duration: Seconds.

Behaviour	Measure	Phases (Mean/SE)			Post-hoc comparison		
		P1	SF	P3	P1-SF	SF-P3	P1-P3
**Gaze**	Frequency	6.46 (0.38)	5.13 (0.38)	6.26 (0.40)	< .001	.008	1.00
	Duration	16.58 (1.23)	11.66 (1.10)	14.20 (1.01)	< .001	.06	.10
**Distressed face**	Frequency	0.38 (0.08)	0.58 (0.10)	0.94 (0.15)	.11	.03	.001
	Duration	3.66 (1.24)	5.17 (1.02)	9.38 (1.85)	.81	.06	.02
**Crying**	Frequency	0.05 (0.02)	0.24 (0.07)	0.51 (0.11)	.01	.002	< .001
	Duration	0.65 (0.31)	6.36 (1.84)	7.72 (1.76)	.004	1.00	< .001
**Sleeping**	Frequency	0.12 (0.07)	0.19 (0.06)	0.16 (0.06)	.23	1.00	1.00
	Duration	0.95 (0.46)	4.16 (1.38)	4.15 (1.72)	.02	1.00	.11
**Sucking**	Frequency	1.38 (0.15)	1.22 (0.13)	1.54 (0.17)	.49	.15	1.00
	Duration	10.47 (1.38)	10.17 (1.28)	12.83 (1.58)	1.00	.25	.55

There was also significant main effect of the experimental manipulation on the duration of gaze (F_2,166_ = 9.91, p<0.001, ηp^2^ = .16). Babies significantly decreased the duration of the eye contact from P1 to SF and had a tendency to increase it again from P1 to SF and to P3. (See Table 2).

#### Distressed face: Frequency and duration

There was a significant main effect of the experimental manipulation on the frequency of the distressed face (F_1.76,146.34_ = 9.32, p<0.001, ηp^2^ = .10). Babies significantly increased the frequency of this behaviour from SF to P3 and overall, from P1 to P3. The change between P1 and SF was not significant. (See Table 2)

There was also a significant main effect of the experimental manipulation on the duration of the distressed face (F_1.67, 138.56_ = 5.74, p = .004, ηp^2^ = .07). Babies showed a continuous increase of the duration of the distressed face throughout the three phases. They had tendency to increase the duration of this behaviour from P1 to SF and then a showed significant increase between P1 and P3. (See Table 2)

#### Crying: Frequency and duration

There was a significant main effect on the frequency of crying (F_1.60,132.93_ = 16.02, p<0.001, ηp^2^ = .16) that significantly increased from P1 to SF, and then further significantly increased from SF to P3. The frequency of crying was significantly higher in P3 compared to P1 (See Table 2).

There was a significant main effect on the duration of crying (F_2,166_ = 10.35, p<0.001, ηp^2^ = .11) that significantly increased from P1 to SF, and remained high in P3. The duration of crying was significantly higher in P3 compared to P1. (See Table 2).

#### Sleeping: Frequency and duration

There main effect on the frequency of sleeping was not significant (F_1.47,122.22_ = 1.04, p = 0.36.

There was, however, a significant main effect on the duration of sleeping (F_1.64,136.24_ = 4.43, p = 0.02, ηp^2^ = .05). Babies significantly increased the duration of sleeping from P1 to SF. The change between SF and P3 was not significant. (See Table 2).

#### Sucking: Frequency and duration

The frequency or the duration of the sucking (F_1.67,138.44_ = 2.22, p = 0.11.; F_1.79,148.29_ = 1.79, p = 0.17) had not been affected by the experimental manipulation (See Table 2).

### 3. The effect of the repeated SFP

#### Gaze: Frequency measures

Repeated Analysis of Variances (ANOVAs) were conducted to investigate the effect of the repeated administration (First/Second) and the experimental phases (P1, SF, P3) on the change of the frequencies of the behaviours of the babies.

The frequency of the Gaze behaviour was significantly affected by the interaction of repetition and experimental phases (*F*_2,166_ = 7.26, *p* = 001, η_p_^2^ = .08). While during the first administration of the SFP babies reduced their gaze from P1 to SF, they recovered their gaze by P3.

When compared the same phases across the repetitions, the frequency of the eye-contact was comparable between P1 in the first and in the second administrations, in the SF in the first and second administration, while eye-contact in P3 was significantly higher in the second administration, thus the result was specific to the recovery of the eye-gaze in P3 in the second administration.

**Fig 1:**
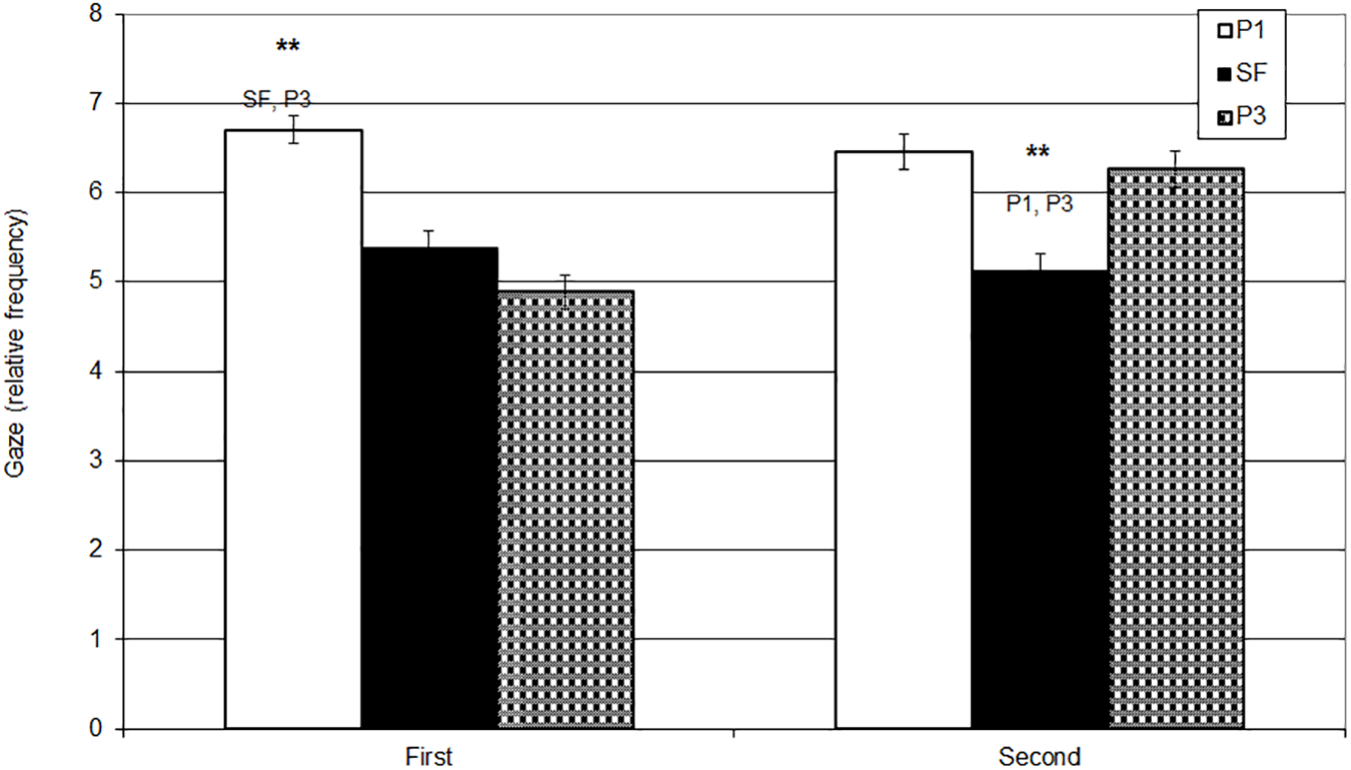
Mean gaze frequencies (with SE) during P1, SF, P3 at the first and the second administration of the SFP. Y axis: Gaze Frequency (rate/minute). X axis: Administration. ** < .01.

As described in the previous analyses, cross-comparing the phases during the first administration of the SFP, babies significantly reduced their gaze from P1 to SF, and kept it low throughout, with a significant decrease from P1 to P3. In the second repetition, however, babies significantly decreased the duration of the eye contact from P1 to SF but increased it again from P1 to P3. (See Table 3, Fig 1).

**Table 3:** Changes of the frequencies and durations of gaze behaviour over P1, SF and P3 in the first and the second experiments. Results of the post-hoc analysis adjusted with Bonferroni method for multiple comparisons. Mean for Frequency: Rate/minute, for Duration: Seconds.

Behaviour	Experiment	Measure	Phases (Mean/SE)			Post-hoc comparison (p)		
			P1	SF	P3	P1-SF	SF-P3	P1-P3
**Gaze**	First	Frequency	6.70 (0.32)	5.38 (0.38)	4.89 (0.37)	< .001	.43	< .001
	Second		6.46 (0.38)	5.13 (0.38)	6.26 (0.40)	< .001	.008	1.00
	First	Duration	16.88 (1.10)	12.68 (1.16)	10.72 (0.96)	.002	.16	< .001
	Second		16.57 (1.23)	11.66 (1.10)	14.20 (1.01)	< .001	.06	.10

#### Gaze: Duration measures

The duration of the Gaze behaviour was significantly affected by the interaction of repetition and experimental phases (*F*_2,166_ = 4.66, *p* = .011, η_p_^2^ = .053).

When cross-comparing the three stages, P1, SF and P3 between the first and the second experiments, the results of the post-hoc comparison showed that both the frequency and the duration measures were significantly different in P3 but were statistically comparable in P1 and in SF.

**Fig 2:**
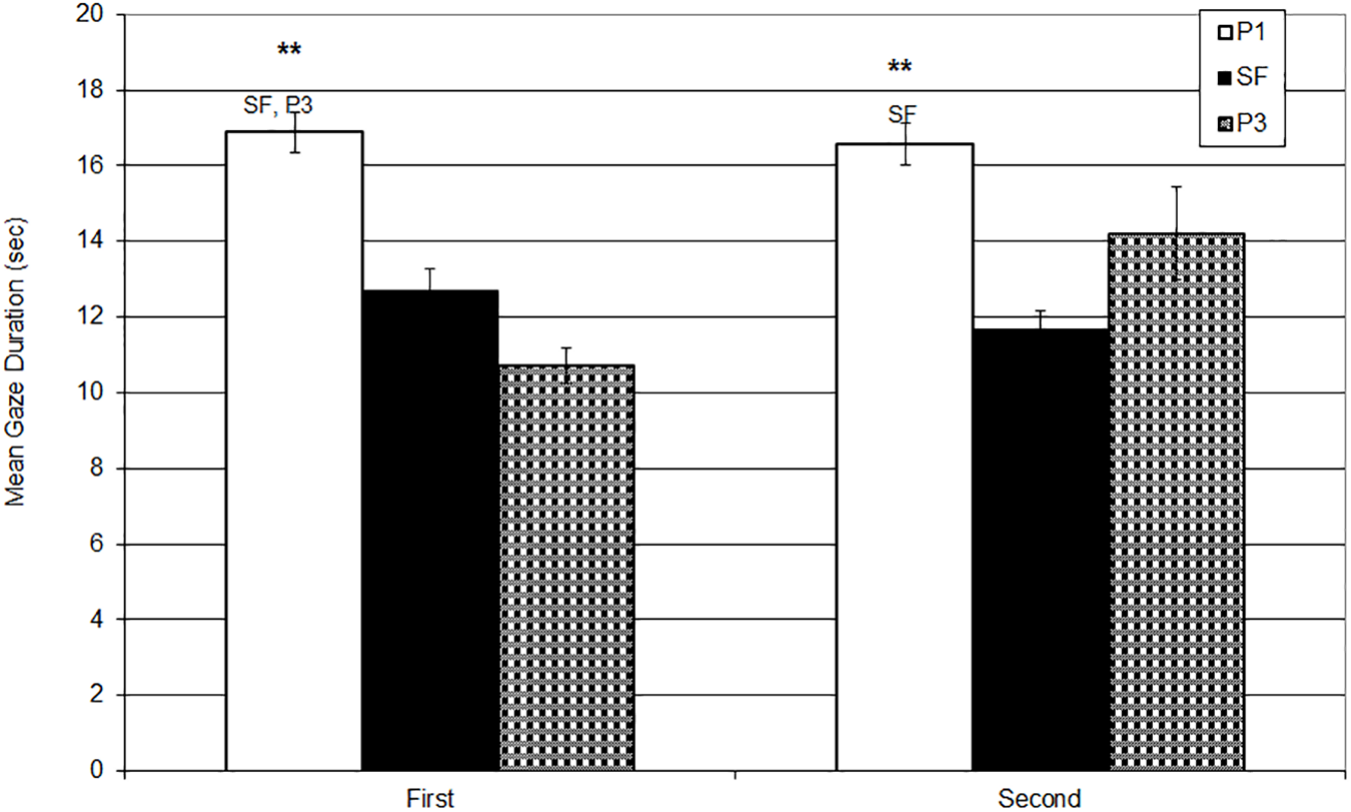
Mean Gaze Durations (with SE) during P1, SF, P3 at the first and the second administration of the SFP. Y axis: Mean Gaze Duration (sec). X axis: Administration. * < .05, ** < .01.

As described in the previous analyses, cross-comparing the phases during the first administration of the SFP, babies significantly reduced their gaze from P1 to SF, and kept it low throughout, with a significant decrease from P1 to P3. In the second repetition, however, babies significantly decreased the duration of the eye contact from P1 to SF but had a tendency to increase it again from P1 to SF and to P3. (See Table 4, Fig 2)

**Table 4:** Post-hoc comparison of the frequencies and the durations of the gaze behaviour across the phases of the first and the second experiment.

**Behaviour**	**Measure**	**Experiment**	**Post-hoc comparison of Phases (p)**		
**Gaze**	Frequency		P1	SF	P3
		First	.52	.56	.002
		Second			
	Duration	First	.83	.45	.005
		Second			

The repeated experience did not affect the frequency changes of distressed face (*F*_1.65,136.91_ = .49, p = .58), crying (*F*_1.28,105.99_ = .02, p = .98), sleeping (*F*_1.57,130.30_ = 1.75, p = .18.), sucking (*F*_2,166_1.46, p = .24) responses over the three phases.

Similarly, the repeated experience did not affect the duration changes of distressed face (*F*_2.166_ = .97, p = .38), crying (*F*_2.166_ = .03, p = .97), sleeping (*F*_1.76,145.83_ = 0.87, p = .42), sucking (*F*_2,166_ = 1.77, p = .17) responses over the three phases.

**Fig 3:**
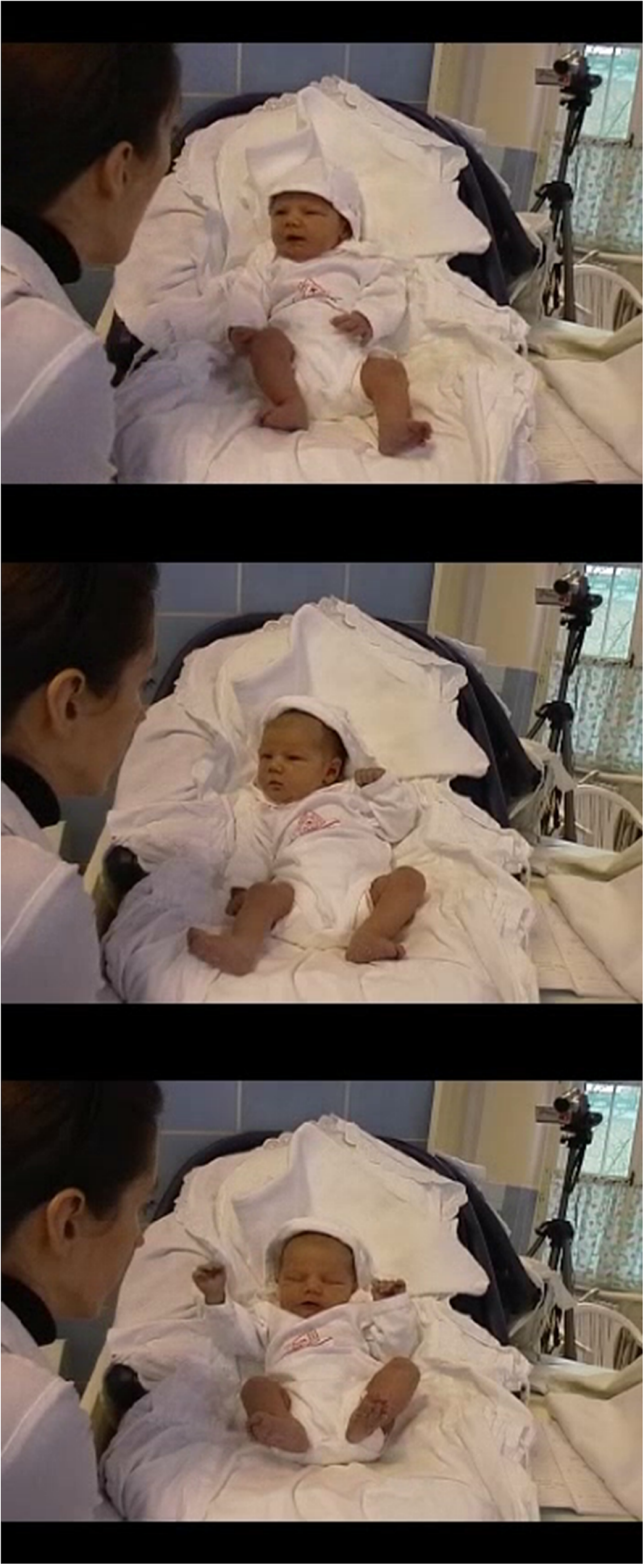
a,b,c Illustration of a neonate’s response to the Still Face Procedure. Fig 3.a P1. Fig 3.b.the start of the SF. Fig 3.c. 20 seconds after the start of the SF.

For illustration of the response, see Fig 3A, 3B and 3C

## Discussion

The results replicated the finding that newborns as young as a few hours old sensitively react to a communication disturbance modelled by the SFP. The behavioural responses measured in this study were similar to our earlier study with newborns [3] and to those with older infants [1].

The present study reported both frequency and duration analyses of eye contact as a measure of approach and avoidance, distressed face and crying as measures of distress, and sleeping and sucking as measures of self-regulation. The frequency measures indicate the effort babies put into achieving a behavioural state, while the duration variables reflect the length of time the newborns spend in a certain state and, thus, often describe different aspects of the behaviour [21, 22].

In this study, in the first administration of the SFP, the babies significantly decreased both the frequency and duration of eye contact in the still-face phase and showed a tendency to further decrease the duration of eye contact from the still-face to the reunion phase. This pattern of change suggests continued effort by the baby to withdraw from the situation and maintain an averted gaze. The pattern is also similar to the results reported in our earlier study [3] where newborns significantly decreased the frequency of gaze from P1 to SF and kept it low from SF to P3, significantly lower than in P1.

The frequency of the distressed face significantly increased from the first phase to the still-face period, while the duration increased more gradually. This pattern signals self-regulatory efforts by the babies. Even though they became increasingly distressed, they tried not to stay in this state for long; hence, the fast increase in frequency but slow, gradual increase in duration. The outcome of this self-regulatory process is probably either a subsequent episode of distressed face or crying, or an attempt to shut down and sleep, as the duration of all three behaviours only gradually increased through the still-face phase into the reunion period.

The increase in sleep as a response to the SFP is seemingly contradictory with a concurrent increase of distressed face and crying. Only further time-series analyses could provide descriptive and explanatory models of the relationship between these behaviours. Given the long temporal window (three-minute-long phases), it is possible that the behaviours are sequential within the same newborn and are the result of rapidly changing states as a response to a stressful situation. It is also possible, however, that a subgroup of newborns react with sleep; the subgroup is large enough to produce a significant result for the entire sample; and individual differences exist in how newborn infants respond to interpersonal stress [23]. Sleep is a fundamental, brainstem level regulation of arousal [24] that newborns effectively use to regulate their own level of stimulation [25]; therefore, it is not surprising that they react to the stress of the SFP with an increase in sleep states.

### SFP in neonates

Overall, the analyses suggest that the reactions to the SFP were confirmed and were comparable with those reported earlier with neonates [3] and older infants [1, 6, 16].

The analyses also showed a similar pattern in the newborns’ reactions to the SFP in the second administration. The frequency and the duration of the distressed face significantly increased from P1 to P3. Similarly, the frequency of crying gradually increased throughout all stages, while the duration of sleep increased from P1 to SF and stayed at a high level in P3. The frequency and duration of gaze decreased from P1 to SF, just as during the first administration, but the carry-over effect disappeared, and babies resumed eye contact with a significant increase in frequency and a tendency to increase the gaze duration to P3. In summary, both the first and the second administrations of the SFP in this study resulted in neonates’ reacting with a typical SF effect.

The reasons for the different results in this study and the two previous studies with neonates might be methodological. In their experiment, Bigelow and Power [18] employed variable timing of the three periods, with the baseline interaction lasting for three minutes, the still-face phase lasting for only one minute and the reunion period for two minutes. They found no evidence for the still-face effect at one week of age and even up to three months of age. Although the authors concluded that there is a lack of still-face effect at one-week of age, the results they reported showed a significant, main effect of the phase on gaze, and the post-hoc test revealed a significant decrease in visual attention from the baseline to the still-face phase. The study therefore partially demonstrated a still-face effect with regard to a decrease in visual attention. The other two variables Bigelow and Power [18] measured were smile and vocalization, which are both prevalent behaviours in older age groups but have a low natural baseline frequency in neonates. These variables were suitable for the longitudinal design but their low baseline might explain the lack of results in their youngest age group.

Bertin and Striano’s [15] study also reported a lack of still-face effect in eighteen newborns on gazing and smiling. The results, however, reported a tendency for the gaze of newborns in the SFP to decrease from the baseline to the still-face phase. They also examined smiling and, just like Bigelow and Power [18], found no changes in this behaviour. Furthermore, each stage lasted for only sixty seconds; therefore, the procedure did not give enough time for newborns with their slower social and perceptual responsivity [26, 27] to fully process and react to the situation. Tronick et al. [1] suggested that three-minute-long phases would be more appropriate to accommodate the infants’ reaction times, and the study by Nagy [3] employed three-minute-long phases. Methodological differences therefore could account for differences among the earlier findings.

### Possible explanations: Fatigue, habituation, self-regulation

#### Fatigue

It is important to note that the results of Nagy’s earlier study [3] could be explained by the effect of fatigue. Nagy [3] employed a control group of 33 newborn infants who were subjected a continuous communication paradigm of comparable length but without the disruption of the still-face phase. When the results of the two groups were compared, the group that was subjected to the SFP showed responses of decreased gaze, increased distressed face and crying and increased sleep during the still-face phase with a carry-over effect of these changes into the reunion period. There were, however, no changes in the control group.

When we cross-compared the three phases of the SFP across the two experiments, we found that the newborns reacted with comparably robust still-face effects both times during the still-face phases. Their behaviour in the reunion period, however, was different. The second time they increased instead of decreased the duration of the gaze, and did not decrease their behaviour. This pattern suggests an active effort rather than fatigue. The results measured in that study and in this study therefore are not only consistent but also could not be attributed to and explained by fatigue.

#### Habituation

It is possible that the changes at the second administration of the SFP could be explained by habituation. Habituation is a basic adaptation process in which a stimulus no longer elicits an emotional response [28]. The SFP however, elicited the same emotional response both times when the babies were exposed to it. The behaviour in the second administration of the SFP started to change compared with the first administration only in the reunion phase. The change involved a potentially adaptive response: an increase in gaze. The fact that the duration and the frequency of the behaviour increased instead of decreased in the reunion phase argues against a simple habituation explanation.

While babies did not habituate to the situation, there was a difference in the pattern of the changes in gaze behaviour during the reunion periods. While in the first administration of the SFP the babies displayed the typical carry-over effect, both in the frequency and the duration of their gaze behaviour, and continued to avert their gaze from the experimenter even after communication was resumed, in the second administration the babies recovered their gaze in the reunion period. Additionally, when cross-comparing the three phases between the first and the second administrations, the frequency and the duration of the gaze were comparable in the first stage and in the still-face phase. This means that from the same baseline levels the babies averted their gaze the second time as often and as long as they did the first time during the still-face phase; therefore, there was no difference in how stressful the still-face phase might have been to them. There was, however, a significant difference in both the frequency and the duration of their gaze behaviour in the third, reunion phase, when the babies significantly increased their gaze from the still face to the reunion periods in the second administration of the SFP instead of continuing to decrease it, as they did in the first administration.

#### Self-regulatory model

If babies habituated to the situation, their response to the still-face phase would have been more blunted, both compared with the first stage and when cross-comparing the still-face phase between the first and the second experiments. The babies’ comparable pattern of reaction to the still-face in the first and the second administration does not support a habituation mechanism or a random response. The responses were stable and consistently similar the first and the second time, suggesting that they perceived the situation as stressful.

It maybe speculated that babies ‘learnt’ from the first administration of the SFP that the interaction partner would resume communication after the stressful disturbance and that this expectation helped them to more efficiently regulate their own recovery from the distressed state after the disturbance was over.

Gaze aversion is one of the more powerful interpersonal regulatory behaviours, especially for the neonate whose repertoire to approach and withdraw is limited. Under- or over-stimulated young infants utilize gaze aversion to reduce heart rate [29], and gaze aversion has been found to effectively decrease distress [30]. The phase for which the repeated experience led to a change in gaze behaviour was the reunion period. Previous studies reported a carry-over negative affect [1], an increase in positive affect and attention [7] and a carry-over effect in gaze aversion [3] in the reunion period. A relative decrease in heart rate has also been reported in the reunion period [31–33], although Moore and Calkins [34] found an increase in heart rate in this phase. While the reunion effects are somewhat varied, it is clear that this period is not a smooth transition from disturbance to re-engagement but a phase when complex regulatory processes take place.

As with their imitative [26] responses, it is likely that regulatory processes in the neonate are prolonged compared with older infants, resulting in a long carry-over period, while gaze behaviour recovers faster and mediates the process of recovery. The recovery of eye contact in the repeated SFP helps babies to utilize the availability of a responsive caretaker and to recover faster from behavioural and physiological distress. Indeed, when Haley and Stansbury [35] measured maternal responsiveness during the still-face situation, infants of more sensitive and responsive mothers showed better recovery during the reunion period compared with infants of less sensitive mothers.

It has already been suggested that newborns are capable of actively controlling and regulating their own states, and by controlling their states, they can control their environment, such as by shutting down unwanted noises and stimulation [36, 37]. Tronick’s Mutual Regulation Model (MRM) suggests that infants and their mothers form a dyadic system in which they mutually co-regulate their behaviours [38]. Similarly, our earlier studies suggested that early neonatal abilities are embedded in the first dialogues [39]. The MRM model also assumes that the success of this mutual co-regulation depends on the mother’s and infant’s abilities to regulate their own homeostatic, affective states. It thus may be speculated that the change in newborns’ behaviours in the second administration reflects an ability to regulate their state through dyadic co-regulation with an interactive partner.

#### The contingency model

The results may also be interpreted with newborns’ preference for contingent responses in interactions. During the SFP, such contingency is lost. In experimental situations, when a mother’s behaviour is played back to her infant using a double-video system [16], and when the replay is non-contingent, this is enough to confuse and upset 6- to 12-week-old infants [16, 40, 41]. When contingency is restored through live play, these infants are happy to re-engage with their mothers. Although these studies were carried out with older infants, they demonstrate that a lack of contingency elicits a distress reaction similar to the SF response. It might be that, during the reunion period, infants find the resumption of the contingent response to be rewarding. As a consequence, they might be motivated to adapt during stressful interpersonal experiences by exhibiting behaviours that could speed up the recovery of contingency and thus reduce their distress.

Because of the quantitative experimental approach of our study, we have not described frame-by-frame case examples. It was, however, commonly observed in the sample that the babies took brief back-and-forth glances at the experimenter, even during the SF phase, even when they were distressed. They showed occasional ‘checking-like’ glances, seemingly coordinated with their arousal and stress levels. This behaviour might become more common the second time the SPF is administered, but a qualitative description of the cases would add to our further understanding of the neonates’ response patterns.

#### Repeated administration of SFP

Two previous studies used a double still-face episode in a form of an A-B-A-B-A design in which the two still-face periods were separated by a period that served as both the reunion after the first still face and a baseline for the still-face phase that immediately followed. Haley and Stansbury [35] used the procedure to examine the behavioural and psychophysiological self-regulatory abilities of five- to six-month-old infants of mothers with high and low sensitivity. The SFP was effective both times; infants did not habituate to the stress of the still-face phase. Although the planned comparisons were not significant, the authors suggested that the infants of highly sensitive mothers were less upset in the second reunion than in the first. Their study found no such changes with regard to gaze, but the data from Haley and Stansbury’s [35] study did not show the typical carry-over decrease in gaze in the first reunion period. Infants of low sensitivity mothers, however, further increased their heart rate in the second reunion period, and infants of highly sensitive mothers reduced their heart rate by the second reunion; however, there were no such differences in the first reunion in which both groups resumed their heart rates to the baseline level. The study might be unusual given the apparent lack of carry-over effect in the measured variables, but it demonstrates how repeated interpersonal stress challenges an infant and results in changes in self-regulatory strategies depending on the infant’s ability to interact with the available social environment–in their case the highly or less sensitive mother.

Hsu and Jeng [42] adopted Haley and Stansbury’s [35] design to test preterm (24–34 gestational age [GA]) and term (38–41 GA) infants’ social responses at two months of corrected age. The results showed a decrease in gaze over the experiment, with a carry-over effect in the first reunion period, a further gradual decrease in the second still-face phase and a tendency to increase gaze in the second reunion phase. The duration of negative affect, however, showed a significant, steady increase over the entire procedure, indicating no habituation. Overall, both experiments repeatedly using the still-face phase suggest that infants do change and adapt their regulatory responses with a second exposure to social stress.

### Possible issues and limitations

#### Can newborns remember the first administration?

It can be questioned whether neonates in their first day of life remember a procedure after an average delay of one day. The comparable responses the first and the second SFP experiences could be attributable to the fact that they did not remember the first administration and responded to the second as if it were novel. Perhaps only the pattern of changes in gaze behaviour from the still-face phase to the reunion period contradicts this assumption. Although some researchers have reported memory in newborns of only a few minutes [43–46], Keen, Chase and Graham [47] found evidence for habituation extending over 24 hours as a response to auditory stimuli. Newborns’ memories of the sounds were indexed by a decrease in heart rate acceleration to the repeatedly administered stimuli, and on the second day the stimulation elicited a smaller cardiac acceleration compared with age-matched control newborns who received the stimuli only on day two but not on day one. Swain et al. [48] found behavioural evidence in two- and three-day-old newborns, indexed by head-turning, of memory of auditory stimuli for over 24 hours. Speech sounds presented in two sessions a day apart elicited habituation, indexed by a decrease in head turning towards the sound and an increase in head turning away from the sound on the second day in newborns who heard the same words both days, but not in the control group that heard a different word nor in the control group that heard neither of the words on day one. Even pre-natal auditory memories have been found to be remembered postnatally. Newborn infants who were presented a particular speech passage in the last six weeks of gestation preferred the familiar passage over a novel one [49].

Newborn infants remember not only auditory but also visual stimuli. Bushnell [50] tested 72-hour-old newborns’ preference for the face of the mother, and newborns maintained their preference for the mother opposed to the face of a stranger, even if the last exposure to the mother had been over 15 minutes previously. Werner and Siqueland [51] reported visual memory for two days with three-week-old infants, while Bushnell et al. [52] reported 24-hour delayed recognition of shapes and forms. It is, therefore, in theory, possible that in the current study, experience, especially of a stressful event, affected newborns’ behaviour one day after the first administration of the SFP.

#### The role of the experimenter

It is worthwhile to note that in the current study an experimenter and not the mother interacted with the newborn. While many of the reports using the SFP employ the mother, a very large number of papers employ a stranger. When the effect of the experimenter was directly compared, Lamb et al. found that infants from one month of age showed more orientation and initiation towards the stranger [11], and the differences in how they behaved with the stranger and the mother affected the baseline and reunion phases, while the infants’ behaviours in the still-face phase were comparable between the two types of interactive partners. This means the still-face effect is less pronounced with mothers and stronger with a stranger experimenter [53], and this in part could explain why the reunion phase is the most variable with regard to potential adaptive regulation over repeated experience.

#### Control group

A relative limitation of the study was the lack of a control group. One reason for not employing a control group was that our earlier experiment [3], which used same methodology and the same outcome variables, found that a control group showed no behavioural changes over comparable three times three-minute-long periods. Thus, there was no reason to expect that a control group would provide phase-specific responses the second time when it did not the first time.

Further studies, however, should not only try to replicate the findings but also include a control group in case a naturally occurring decrease in gaze occurs at three to six minutes, followed by an increase at six to nine minutes for the second, but with no change the first time without an intervention.

#### Obstetric factors

A relative limitation of the study is that obstetric factors have not been considered in the statistical analysis. There are four studies in the literature that have examined the effect of very low birth weight, prematurity and responses to the SFP. Segal et al. [54] administered the SFP to sixteen preterm (24–36 weeks GA at birth) and 18 full-term (38–42 weeks GA at birth) infants at seven months of age and found that both groups showed the typical still-face effect and responded to the situation comparably in all three stages, with the difference that preterm infants displayed fewer “big” smiles during the still-face phase.

Hsu and Jeng [42] compared the SFP responses of twenty full-term (38–41 weeks GA at birth) and twenty preterm mother–infant dyads (24–34 weeks GA at birth) at two months of corrected age. Both groups reacted with comparable sensitivity to the procedure, but the preterm group became distressed somewhat faster and stayed in the negative affect state for longer.

Jean and Stack [55] examined the SFP responses at 5.5 months of age of forty preterm (mean GA at birth 26.51 weeks) and forty full-term (mean GA at birth 39.51 weeks) infants with their mothers. While both groups reacted to the situation with comparable sensitivity, the full-term group exhibited more self-comforting behaviours in the reunion period. Finally, Montirosso et al. [56] examined the SFP responses of twenty-five preterm (26–36 weeks GA at birth) and twenty-five term infants (37–41 weeks GA at birth) six to nine months after delivery with their mothers. While both groups reacted with the same sensitivity to the situation, the full-term group showed more distancing during the still-face phase, while the preterm group showed more social monitoring during the reunion phase. Taken together, maturity at birth and paediatric risk factors are likely to play a role in some of babies’ reactions to the SFP, but their overall sensitivity is comparable. Each of the above studies studied extremely preterm (<28 weeks GA) and very preterm (28–32 weeks) babies. In the current sample, all but four of the 84 newborns were born full term, while the remaining four were born at 36 weeks GA. The subgroup therefore had too small a GA to be taken into account as a factor. Regarding the babies’ age, the time difference between the first and the second administration was a mean of 1.25 days but that included 2 hours to 4 days difference between the two administrations. Future studies could ensure a more homogenous delay for the second administration.

#### Mode of delivery

Regarding the impact of the mode of delivery on the newborn, Field and Widmayer [57] followed from birth 20 infants born through caesarean delivery (CD) and 20 control infants born through vaginal delivery. There were no differences in the neonatal period as assessed by the Brazelton Neonatal Behavioural Assessment Scale (NBAS) [37]. As part of their longitudinal study assessing the association between mode of delivery and postpartum depression, Garel et al. [58, 59] assessed how the mode of delivery affected the behaviour of the infant in the short and long-term. The study found no differences in mother–infant interactions at any of the points they examined. Gottlieb and Barrett also [60] found no difference in the behaviour of newborns as measured by the NBAS on day two or in mother–infant interaction at one month postpartum after CD. Neither did Culp et al. find any differences [61] at three months postpartum when analysing the behaviour of mother–infant dyads according to mode of delivery.

Although we did not include mode of delivery as a factor in the statistical analysis, further studies should explore the question and include obstetric and paediatric variables.

#### Skin-to-skin contact

Bigelow and Power [18] suggested that early skin-to-skin contact facilitates social sensitivity to the SFP. In their study, they asked twenty-eight mothers to engage in skin-to-skin contact with their newborns for six hours in the first week and compared the infants’ reactions to the SFP with those who did not receive enhanced skin-to-skin contact. Those with early skin-to-skin contact started to show reactions to the SFP at one month of age, while the control group did so only at two months of age. The differences remained at three months, with the experimental group engaging in more ‘bidding’ behaviour with their mothers during the procedure. The current study collected no data on the skin-to-skin contact the mothers engaged in with their newborns, but further studies should take this powerful variable into account.

Of the over 80 studies Mesman et al. examined in their systematic review, which used the SFP as part of their design [6], only eleven studies measured the mother’s behaviour in some way and related it to the infants’ responses. Two of these studies focused on the reunion period specifically. Rosenblum et al. [62] measured maternal and infant behavioural responses in the reunion period and found that maternal positive affect and involvement predicted infants’ positive affect, while maternal intrusiveness predicted the infants’ avoidance. Haley and Stansbury [35] averaged maternal responsiveness across the three periods and found that moderate and high responsive mothers’ infants reacted with increased gaze aversion during the still-face phase and showed better recovery during the reunion.

Since the present study used a detailed frame-by-frame behavioural analysis with five-millisecond (frame) accuracy for the entire period of the SFP lasting nine minutes for 84 newborns (performed twice), this study had no resources to analyse the experimenter’s behaviour. Also, it was not the mother but the experimenter who participated in the dyad; therefore, it was assumed that her style remained the same with every baby throughout. Still, further quantitative objective measures would be needed to rule out any systematic bias over time or in relation to certain reactions by the babies during the first and the reunion periods.

#### Maternal factors

The effect of maternal factors on newborns’ reactions to the SFP could also be investigated in the future. Given that mothers with postpartum depression have marked communication impairment [63, 64] and that the infants of depressed mothers generalize their behaviours even with non-depressed strangers [65], maternal depression could affect infants’ responsivity to communication and communication disturbances in the SFP [6].

Interestingly, the results in the literature are inconclusive with regard to the effect of a mother’s postpartum depression on her infant’s reaction to the SFP.

A few studies have reported the lack of a clear relationship [54, 62, 66]: that the difference in a mother’s behaviour did not translate to a measurable difference in her infant’s behaviour during the SFP. However, Weinberg et al [32, 67] have reported an increased negative affect in the sons of postpartum depressed mothers in the reunion period at three months of age. Another group of studies has reported a decrease in the SF effect in infants of postpartum depressed mothers [68, 69]. For example, studies have reported that both male and female infants of depressed mothers were less sensitive to the SFP, showed fewer distress behaviours during the SF phase, had less interaction during the reunion phase and exhibited fewer negative and positive behaviours even if they were more active than infants of non-depressed mothers. Three-month-old infants of mothers with a history of depression, however, have shown an increase in negative affect [70], unlike the decrease reported in the previous studies. Further studies, therefore, should consider the role of maternal factors, including psychopathology, socio-economical status and parity when evaluating the effect of the SFP on newborn infants.

## Conclusion

In conclusion, neonatal infants not only showed robust reactions with a carry-over effect in the first administration of the SFP, but also similarly robust reactions a second time in the still-face phase of the second administration of the SFP. When comparing the first and the second administration, their responses began to change in the second reunion phase, the carry over effect for gaze, but not for other distress behaviours, disappeared the second time, that allowed us to speculate whether they adaptively utilized the socially interactive partner in their own recovery. Such interpretations, although must be considered with caution due to the multiple limitations discussed above, could be consistent with an adaptive self-regulatory explanation.

The active involvement of the infant in regulating her own development is still underestimated [36]. Neonates’ ability to proactively initiate interaction within an imitative context [71, 72] has frequently been described as evidence of their role in shaping their interpersonal interactions. Infants of sensitive mothers were reported to show more approach behaviours such as gazing and smiling [73] and to have a lower level of negative affect, more self-soothing [6, 74] and increased seeking of the mother’s attention [33, 62]. These responses might signal that infants actively utilize the mother as a regulatory source in times of stress.

Overall, we might speculate that newborns are able to actively regulate their own states within their social interactions. They could do so by changing their behaviours in an adaptive way, shaped by their previous experience. This pro-active, self-regulatory control could allow newborns to adapt their behaviours in the context of the interaction. We can make such assumptions, but whether such a phenomenon is common in the everyday interactions of newborn babies outside the laboratory setting has yet to be explored.

Further studies, taking into account the above limitations could approach understanding newborn behaviour as a dynamic, interactive process in which the neonate, based on her previous experiences, actively uses and possibly even initiates increasingly efficient self–other regulatory processes.
